# Supplementary material for: Effect of Oxidative Damage on the Stability and Dimerization of Superoxide Dismutase 1
Source: Biophys J. 2016 Apr 12;110(7):1499–509. doi: 10.1016/j.bpj.2016.02.037 (PMC4833831; doi:10.1016/j.bpj.2016.02.037)
Supplement: Document S1. Supporting discussion and Fig. S1 [file mmc1.pdf]

Biophysical Journal, Volume *110*

Supplemental Information

Effect of Oxidative Damage on the Stability and Dimerization of Superoxide Dismutase 1

Drazen Petrov, Xavier Daura, and Bojan Zagrovic

Biophysical Journal

Supporting Material

Effect of Oxidative Damage on the Stability and Dimerization of Superoxide Dismutase 1

D. Petrov,[1] X. Daura,[2,3] and B. Zagrovic[1,*]

[1]Department of Structural and Computational Biology, Max F. Perutz Laboratories, University of Vienna, Vienna, Austria; [2]Institute of Biotechnology and Biomedicine, Universitat Autònoma de Barcelona, Bellaterra, Spain; and [3]Catalan Institution for Research and Advanced Studies (ICREA), Barcelona, Spain

Comparison of calculated and experimental changes in free energy upon ALS-related mutations

Using the same approach as for oxidative modifications, we have calculated changes in free energy upon three ALS-related mutations affecting residues on the homodimer interface (A4V, C6A and I113T).

The calculated free energy changes of A4V and I133T mutations match well the experimental data with all of the changes having the correct sign and deviations from experiment exhibiting a median of 4.3 kJ/mol and an average of 4.7 kJ/mol (Figure 1). The only major outlier is seen in the case of the monomer-to-dimer transition for the A4V mutant (9 kJ/mol). Finally, C6A mutation shows marginal stabilization which is in agreement with experimental findings that this mutation does not affect the stability of SOD1 (Figure 1). Experimental data were taken from (1) (A4V), (2) (C6A) and (3) (I113T).

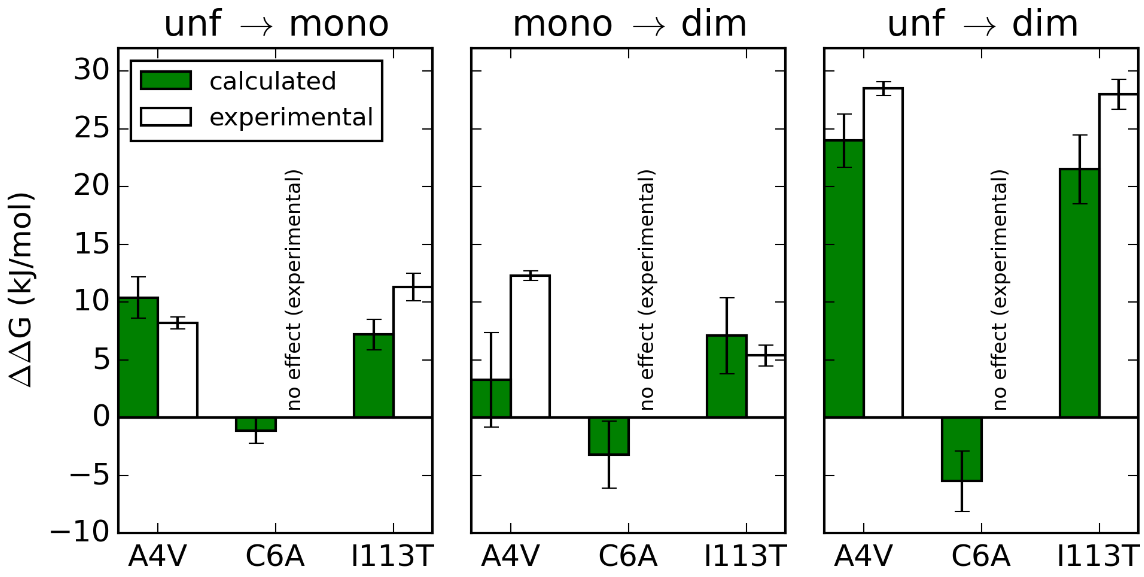

Figure 1. Comparison of the calculated and experimental changes of the free energy of SOD1 monomer folding (left), dimerization (middle) and folding and dimerization (right). Note that the free energy of folding and dimerization was calculated as: $\Delta\Delta G^{nat\to oxi}_{unf\to dim} = 2\Delta\Delta G^{nat\to oxi}_{unf\to mono} + \Delta\Delta G^{nat\to oxi}_{mono\to dim}$. “No effect” refers to experimental finding where no measureable effect of the mutation was found.

Supporting References

1. Svensson, A. K. E., O. Bilsel, C. Kayatekin, J. A. Adefusika, J. A. Zitzewitz, and C. R. Matthews. 2010. Metal-free ALS variants of dimeric human Cu,Zn-superoxide dismutase have enhanced populations of monomeric species. PLoS ONE 5:10.
2. Lindberg, M. J., L. Tibell, and M. Oliveberg. 2002. Common denominator of Cu/Zn superoxide dismutase mutants associated with amyotrophic lateral sclerosis: Decreased stability of the apo state. Proc. Natl. Acad. Sci. U.S.A. 99:16607-16612.
3. Vassall, K. A., P. B. Stathopulos, J. A. O. Rumfeldt, J. R. Lepock, and E. M. Meiering. 2006. Equilibrium thermodynamic analysis of amyotrophic lateral

sclerosis-associated mutant apo Cu,Zn superoxide dismutases. Biochemistry 45:7366-7379.
