# Supplementary material for: Effect of Oxidative Damage on the Stability and Dimerization of Superoxide Dismutase 1
Source: Biophys J. 2016 Apr 12;110(7):1499–509. doi: 10.1016/j.bpj.2016.02.037 (PMC4833831; doi:10.1016/j.bpj.2016.02.037)
Supplement: Document S2. Article plus Supporting Material [file mmc2.pdf]

Biophysical Journal
Article

Biophysical Society

Effect of Oxidative Damage on the Stability and Dimerization of Superoxide Dismutase 1

Drazen Petrov,[1] Xavier Daura,[2,3] and Bojan Zagrovic[1,*]
[1]Department of Structural and Computational Biology, Max F. Perutz Laboratories, University of Vienna, Vienna, Austria; [2]Institute of Biotechnology and Biomedicine, Universitat Autònoma de Barcelona, Bellaterra, Spain; and [3]Catalan Institution for Research and Advanced Studies (ICREA), Barcelona, Spain

ABSTRACT During their life cycle, proteins are subject to different modifications involving reactive oxygen species. Such oxidative damage to proteins may lead to the formation of insoluble aggregates and cytotoxicity and is associated with age-related disorders including neurodegenerative diseases, cancer, and diabetes. Superoxide dismutase 1 (SOD1), a key antioxidant enzyme in human cells, is particularly susceptible to such modifications. Moreover, this homodimeric metalloenzyme has been directly linked to both familial and sporadic amyotrophic lateral sclerosis (ALS), a devastating, late-onset motor neuronal disease, with more than 150 ALS-related mutations in the SOD1 gene. Importantly, oxidatively damaged SOD1 aggregates have been observed in both familial and sporadic forms of the disease. However, the molecular mechanisms as well as potential implications of oxidative stress in SOD1-induced cytotoxicity remain elusive. In this study, we examine the effects of oxidative modification on SOD1 monomer and homodimer stability, the key molecular properties related to SOD1 aggregation. We use molecular dynamics simulations in combination with thermodynamic integration to study microscopic-level site-specific effects of oxidative "mutations" at the dimer interface, including lysine, arginine, proline and threonine carbonylation, and cysteine oxidation. Our results show that oxidative damage of even single residues at the interface may drastically destabilize the SOD1 homodimer, with several modifications exhibiting a comparable effect to that of the most drastic ALS-causing mutations known. Additionally, we show that the SOD1 monomer stability decreases upon oxidative stress, which may lead to partial local unfolding and consequently to increased aggregation propensity. Importantly, these results suggest that oxidative stress may play a key role in development of ALS, with the mutations in the SOD1 gene being an additional factor.

INTRODUCTION

Reactive oxygen species (ROS) participate in a large number of different chemical reactions with proteins, leading to modified amino-acid side chains and backbone or even cross-linked and fragmented proteins (1). Importantly, such oxidative modifications have been associated with aging and age-related disorders such as neurodegenerative diseases, cancer, or diabetes (2,3). Additionally, highly oxidized proteins have been found in potentially cytotoxic protein aggregates and amyloid fibrils (4,5). Furthermore, recent evidence indicates that carbonylation, arguably the most studied irreversible oxidative modification, increases the aggregation propensity of proteins and can thus trigger the formation of insoluble protein inclusions by itself (6). However, a direct causal relationship between oxidative stress on the one hand and protein aggregation, aging, and development of late onset diseases on the other is still largely unclear (7). An important and widely studied system for exploring this relationship has been Cu/Zn superoxide dismutase 1 (SOD1) (8,9). This enzyme has been found to associate with 20% of the cases of familial amyotrophic lateral sclerosis (fALS), an age-related neurodegenerative disease, with more than 150 different mutations in the SOD1 gene having been linked with this condition (8,10,11). Moreover, the presence of SOD1 in protein inclusions in motor neurons and astrocytes of fALS patients (12–14) and animal models (15,16) has been observed and documented in detail. Interestingly, SOD1, which breaks down free superoxide radicals as one of the key antioxidant enzymes in human cells, is by the nature of its function as well as its cellular localization exposed to higher levels of oxidative stress than most other proteins. In line with this, it has been suggested that direct oxidative damage of SOD1 may be implicated in the etiology of sporadic ALS (17–23), which accounts for ~90% of all ALS cases. In particular, it has been shown that changes in the

Submitted October 8, 2015, and accepted for publication February 10, 2016.

*Correspondence: bojan.zagrovic@univie.ac.at

Editor: Bert de Groot
http://dx.doi.org/10.1016/j.bpj.2016.02.037

© 2016 Biophysical Society
This is an open access article under the CC BY license (http://creativecommons.org/licenses/by/4.0/).
CrossMark

wild-type pattern of posttranslational modifications of SOD1 or the introduction of unnatural modifications in the wild-type protein are associated with destabilization of the protein or its dimer, leading to misfolding and aggregation (17–23). However, the molecular causes and potential implications of oxidative stress in the development of ALS still remain elusive. In particular, it is not clear to what extent oxidative damage in vivo is an actual cause of SOD1 aggregation or, alternatively, is a downstream consequence of aggregation.

Under native conditions SOD1 forms a stable homodimer and binds zinc and copper ions, which are critical for its dismutase activity as well as its tertiary and quaternary structure formation and stability (20,24,25). Importantly, formation of a homodimer has been shown to prevent SOD1 aggregation and subsequent cytotoxicity (19). The two main mechanisms that have been proposed to explain the toxicity of SOD1 mutants are 1) reduced dismutase activity or increased peroxidase activity leading to an overall increase in oxidative damage of cellular proteins, and 2) formation of insoluble aggregates through a decrease in the stability of SOD1 monomers and/or SOD1 dimer destabilization. Concerning the latter, it has been suggested that it is possibly due to a decrease in binding affinity for copper and zinc ions with a concomitant increase in their cellular levels, which by itself is thought to be neurotoxic (8,20). In a similar fashion, oxidative damage of wild-type SOD1 has been shown to affect the activity of the enzyme as well as result in a reduction in monomer and/or dimer stability and an increase in subsequent aggregation. For example, Chakrabartty et al. have convincingly shown that the oxidation of active-site histidine residues results in the release of the bound, catalytically active metals with subsequent structural changes and nonamyloid aggregation of the protein itself (18,19,26). Moreover, Martins and English have used high-resolution mass-spectrometry to show that oxidative damage to residues Cys146, His71, and His120 predisposes the molecule for misfolding and aggregation (23). Finally, Guareschi et al. have shown that an iper-oxidized form of SOD1 found in sporadic ALS with bulbar onset may exhibit the same toxic mechanisms as mutant SOD1 (27). As a counterpoint to these findings, however, it should be emphasized that oxidized SOD1 has been detected in the brains of individuals afflicted by Alzheimer's and Parkinson's disease as well (28), suggesting that the presence of misfolded and aggregated conformers of the highly abundant SOD1 may be a nonspecific consequence of aging and disease.

In this study, we use molecular dynamics (MD) simulations (29,30) to investigate whether and under what circumstances direct oxidative damage of the SOD1 enzyme could trigger cytotoxicity. In particular, we explore how different oxidative modifications at the homodimer interface affect dimer stability, and we further ask how these modifications modulate the stability of free monomers in solution. Importantly, although significant efforts have been directed at experimentally characterizing the thermodynamic properties of SOD1 wild-type and fALS mutants (31,32), a systematic investigation of oxidatively modified SOD1, i.e., of oxidative "mutations" at the atomistic level, is still in its infancy. With the continued advance of computer power, this problem has become tractable by different theoretical and computational approaches. On the one hand, various efficient semiempirical methods, utilizing force-field- and knowledge-based scoring functions to predict protein stability upon mutation, have been developed (33–35). Although validation against experimental data has shown that such methods correctly reproduce general trends, they often fail in providing a precise quantitative measure of stability (36). In addition, such methods are envisioned for a limited number of canonical mutations, rather than for general purpose calculations including oxidative modifications. On the other hand, perturbation techniques in combination with classical MD represent a rigorous, physically based, and arguably more accurate approach, to estimate changes in conformational free energies upon mutation (37–41). For example, Seeliger and de Groot (37) have successfully calculated thermodynamic-stability differences for 109 mutants of the microbial ribonuclease barnase achieving a remarkable accuracy, with a Pearson correlation coefficient of $R = 0.86$ against experimental data and an average absolute error of only 3.31 kJ/mol, using nonequilibrium fast-growth thermodynamic integration techniques. Furthermore, Lin et al. (38) have performed one-step perturbation calculations to explore the effects of different side-chain substitutions on the folding equilibrium of a hepta-β-peptide, obtaining results in agreement with experimentally available NMR and circular dichroism data.

In our study, we employ the thermodynamic-integration (TI) method (42), one of the most widely used and most thoroughly tested techniques available for the calculation of free-energy differences (43–45), in combination with equilibrium MD simulations to estimate changes in stability of the SOD1 monomer and homodimer upon oxidative damage. Although computationally extremely expensive, TI yields free-energy differences at the limit of accuracy of the force field used, given sufficient sampling (46–48). Using TI, we explore the effect of oxidative modifications of nine residues at the homodimer interface (THR2, LYS3, CYS6, LYS9, THR54, PRO62, CYS111, ARG115, and THR116). We have chosen these particular residues because they are 1) highly susceptible to oxidation, and 2) because of their location, expected to affect the homodimer stability more strongly than others. Namely, out of 46 interfacial residues (all residues of an SOD1 monomer that are within 8 Å of the other monomer in the homodimer structure), we focused on carbonylation of threonines, lysines, arginines, and prolines, and oxidation of cysteines to cysteic acids (Fig. 1), two of the most frequent and important types of oxidative modification found in nature (1–3). In addition, these modifications lead to a major change in local hydrophobicity, a physicochemical variable of key

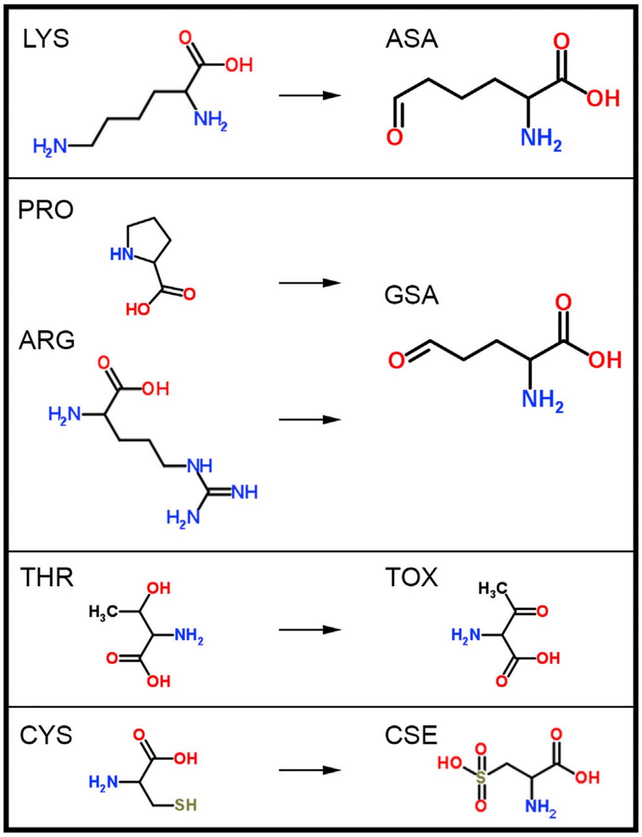

FIGURE 1 Summary of the studied oxidative modifications: 1) lysine to aminoadipic semialdehye (carbonylation), 2) proline and arginine to glutamic semialdehyde (carbonylation), 3) threonine to 2-amino-3-ketobutyric acid (carbonylation), and 4) cysteine to cysteic acid (oxidation) modifications. Note that the chemical structures are shown in their neutral forms, whereas in simulations the protonation states of all residues correspond to the most abundant state in solution at neutral pH. To see this figure in color, go online.

importance when it comes to the stability of biomolecular structures and their complexes (49,50). Our results provide a site-specific, atomic-level picture of the effects of oxidative modifications on SOD1 structural properties.

MATERIALS AND METHODS

Molecular dynamics simulations and free-energy calculations

We have used the TI approach (42) to calculate the impact of different types of oxidative modifications on the stability of SOD1 dimer and monomer. Alchemical modifications from native residues of interest to their oxidized forms, in the context of the folded SOD1 homodimer and monomer or its unfolded state (modeled by a GGXGG pentapeptide, where X stands for the affected residue), were performed by smoothly modifying the force-field parameters from those defining the initial state to those defining the end state. The process was coupled to a parameter λ, ranging from $\lambda = 0$ to $\lambda = 1$, with the end points representing the native and the modified residue, respectively. Starting from a fully stretched pentapeptide or a three-dimensional structure of SOD1 (PDB code 3KH4 (51), using the chains A and B for dimer and the chain A for monomer simulations), each system was solvated in a cubic box filled with explicit Simple Point Charge (52) water molecules, energy minimized, and subsequently equilibrated in three independent copies by gradually increasing the temperature (from 100 to 300 K) over 100 ps and decreasing position restraints on peptide or protein atoms (from 25,000 to 5,000 kJ mol^{-1} nm^{-2}) at constant volume and temperature. An additional equilibration for 20 ps at constant pressure (1 bar) and temperature (300 K) was then performed. Starting from each of the three equilibrated system, three independent, 500 ps MD simulations were run at each of 21 equally spaced λ-points, with two additional λ-points near both ends of the λ range, for a total of 112.5 ns per system. The change in free energy of an alchemical modification was then calculated as the integral of the ensemble average of the derivative of the system Hamiltonian with respect to λ, between the boundaries $\lambda = 0$ and $\lambda = 1$. The integrals were evaluated by the generalized Simpson's rule for nonequidistant nodes using averages over the nine independent simulations at each λ-point, including only the last 150 ps of each 500 ps simulation. The change upon oxidative modification in the stability of SOD1 homodimer ($\Delta\Delta G^{nat\rightarrow oxi}_{mono\rightarrow dim}$) and monomer ($\Delta\Delta G^{nat\rightarrow oxi}_{unf\rightarrow mono}$) were calculated according to the thermodynamic cycle in Fig. 2 as follows:

$$\Delta\Delta G^{nat\rightarrow oxi}_{mono\rightarrow dim} = \Delta G^{oxi}_{mono\rightarrow dim} - \Delta G^{nat}_{mono\rightarrow dim} = \Delta G^{nat\rightarrow oxi}_{dim} - 2\Delta G^{nat\rightarrow oxi}_{mono} \quad (1)$$

and

$$\Delta\Delta G^{nat\rightarrow oxi}_{unf\rightarrow mono} = \Delta G^{oxi}_{unf\rightarrow mono} - \Delta G^{nat}_{unf\rightarrow mono} = \Delta G^{nat\rightarrow oxi}_{mono} - \Delta G^{nat\rightarrow oxi}_{unf}, \quad (2)$$

where $\Delta G^{oxi}_{mono\rightarrow dim}$ and $\Delta G^{nat}_{mono\rightarrow dim}$ are free energies of dimer formation of the oxidatively modified and native SOD1 homodimer, respectively; $\Delta G^{oxi}_{unf\rightarrow mono}$ and $\Delta G^{nat}_{unf\rightarrow mono}$ are an approximation to the folding free energies of the oxidatively modified and native SOD1 monomer, respectively; and $\Delta G^{nat\rightarrow oxi}_{dim}$, $\Delta G^{nat\rightarrow oxi}_{mono}$, and $\Delta G^{nat\rightarrow oxi}_{unf}$ are free-energy changes upon alchemical modification of the folded SOD1 homodimer and monomer, and the unfolded monomer, respectively. Statistical errors were estimated using block averaging and standard propagation of error (53). MD simulations were run using the GROMACS 4.0.7 biomolecular simulation package (54), with the GROMOS force-field 54A7 parameter set (55,56), integration time step of 2 fs, a reaction-field electrostatic scheme with cutoff $r_c = 1.4$ nm and dielectric constant $\varepsilon_{rf} = 65$, and Berendsen thermostat and barostat (57) keeping the temperature at 300 K ($\tau_T = 0.05$ ps) and pressure at 1 bar ($\tau_p = 1$ ps and compressibility $= 4.5 \times 10^{-5}$ bar^{-1}). A soft-core formalism (58) was used to avoid singularities in the potential energy function when removing the nonbonded interactions of atoms, with parameters *sc-*$\alpha = 0.7$ and *sc-power* $= 1$, except for threonine carbonylation for which *sc-*$\alpha = 1.51$ and *sc-power* $= 2$ were used. Using the above free-energy-calculation protocol, the effect of oxidative modifications of nine residues at the homodimer interface (THR2, LYS3, CYS6, LYS9, THR54, PRO62, CYS111, ARG115, and THR116) was explored.

Topology generation

Parameters of the modified residues were obtained from the Vienna-PTM server: as described in (50,59), they were derived in analogy to parameters of canonical amino acids following the parameterization strategy of the GROMOS force field, which tries to reproduce experimental hydration free energies for side-chain analogs, a fundamental physicochemical property in the context of protein folding in an aqueous environment. Hybrid topologies describing the alchemical perturbations were generated in such a way that a minimal number of atoms were perturbed. Exceptionally, in the case of the amino acid proline (PRO) to glutamic semialdehyde (GSA) modification, a dual topology was used, where all the atoms of the PRO residue were perturbed into dummy atoms whereas all GSA atoms

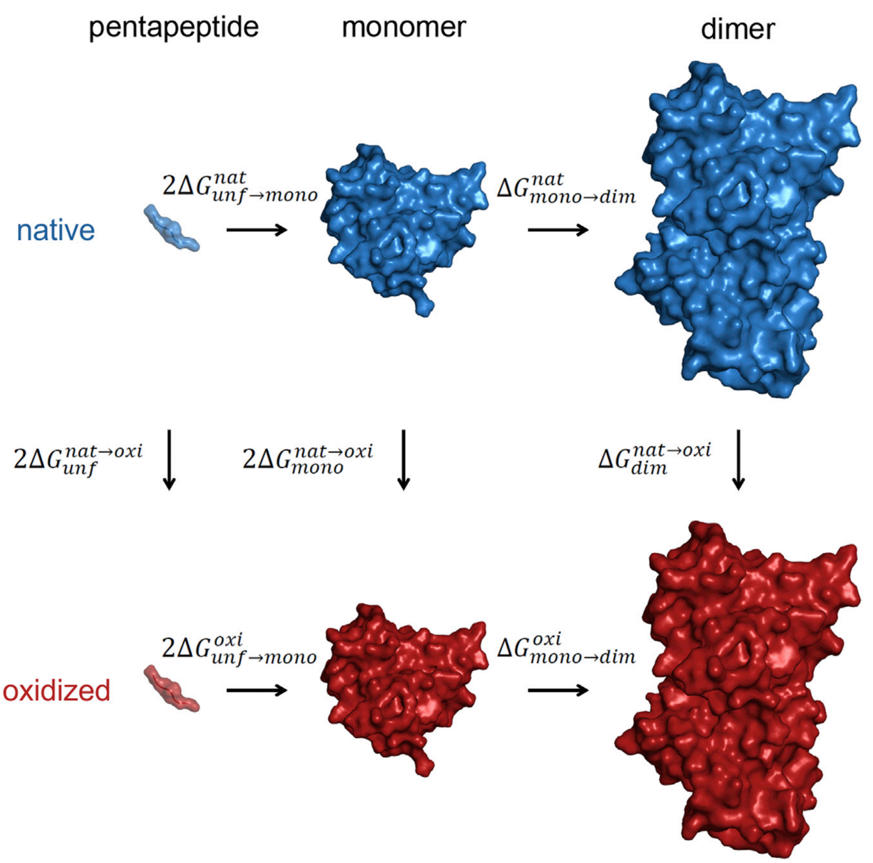

FIGURE 2 Thermodynamic cycle. The relative free-energy difference between the native and the oxidized SOD1 dimer/monomer stability was determined by evaluating the changes in the free energy of an alchemical modification from a native residue of interest to its oxidized form ($\Delta G^{nat\rightarrow oxi}_{dim}$ for an alchemical modification in the dimer, $\Delta G^{nat\rightarrow oxi}_{mono}$ for an alchemical modification in the monomer, and $\Delta G^{nat\rightarrow oxi}_{unf}$ for an alchemical modification in the pentapeptide, i.e., unfolded state), according to the above thermodynamic cycle and Eqs. 1 and 2. To see this figure in color, go online.

were perturbed from dummy atoms into interacting atoms. Note that this ensures that GSA residues derived from both PRO and ARG have the same description in terms of the system's Hamiltonian.

Trajectory analysis

GROMACS 4.0.7 analysis tools (54) were used to analyze simulated trajectories, including the native and oxidized end states. Atom-positional root-mean-square deviation after rotational-translational fitting was calculated with respect to the experimental SOD1 structure (PDB code 3KH4 (51)). Potential energy between a given residue and the rest of the system was calculated according to the GROMOS force-field 54A7 parameter set (55,56), also used for generating the trajectories. Number of charge-charge interactions were calculated as a number of oppositely charged species within a range between 0.3 and 0.6 nm. All reported values are averages over nine independent 500 ps MD simulations. Additionally, three independent 50 ns MD simulations were performed for the native and the oxidatively modified SOD1 molecules, each in the context of the monomer and the homodimer, including oxidized LYS3, LYS9, CYS111, and ARG155 residues. These simulations showed no significant difference with respect to the end-state simulations (data not shown).

RESULTS

Alchemical switching from native to oxidatively damaged SOD1

We have used thermodynamic integration to calculate the free-energy changes associated with the alchemical switching of nine native residues at the SOD1 homodimer interface (THR2, LYS3, CYS6, LYS9, THR54, PRO62, CYS111, ARG115, and THR116) to their oxidatively modified forms. In particular, we have employed a widely used equilibrium approach based on free-energy cycles (Fig. 2). Insufficient sampling is one of the major limitations of the MD method, especially in free-energy calculations for complex systems with rough free-energy landscapes. We have tried to maximize sampling by performing nine independent simulations per λ-point (a total of 112.5 ns for each alchemical perturbation). To estimate the statistical errors of the ensemble averages of the derivative of the system Hamiltonian with respect to λ ($<\partial H/\partial\lambda>$) we have used block averaging (see Materials and Methods). The analysis of the distribution of errors at individual λ-points shows that ~90% of errors are smaller than 10 kJ/mol, with the average over all SOD1 dimer and monomer simulations being 5.2 kJ/mol, comparable with 2RT $\approx$ 5 kJ/mol at room temperature. Additionally, the smooth $<\partial H/\partial\lambda>$ versus λ curves obtained (Fig. 3) allow for an adequate numerical estimation of the integrals (free-energy differences), with all errors below 4 kJ/mol. This suggests that our free-energy calculations are reasonably converged. The only exception to the above is CYS6. Namely, despite the equal degree of sampling as compared with other residues, more than 20% of the errors from

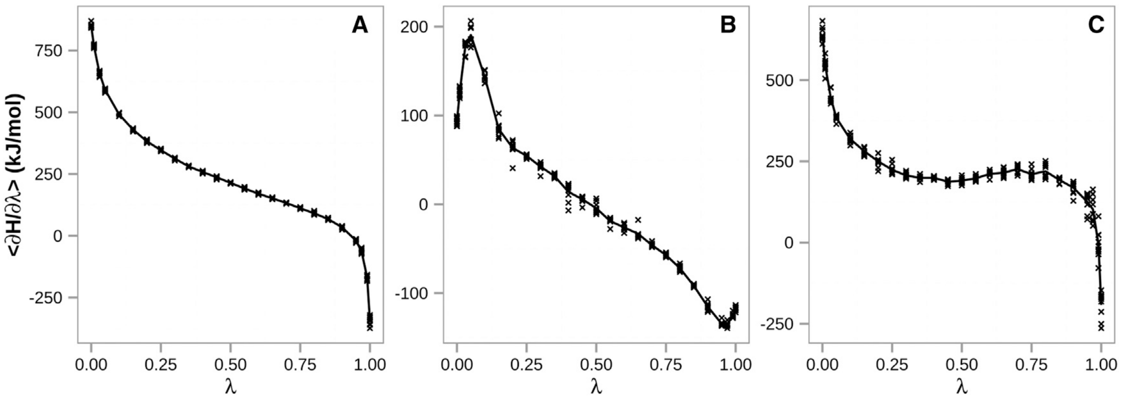

FIGURE 3 Typical <∂H/∂λ> curves. Ensemble average are derivative of the system Hamiltonian with respect to λ shown as a function of λ: (*A*) LYS9, (*B*) THR54, and (*C*) ARG115. Multiple points at a given λ come from independent simulations, with the average curves shown in solid lines.

simulations probing oxidative modification of CYS6 are greater than 20 kJ/mol, with an average of 14 kJ/mol, clearly showing that convergence has not been reached for this system. A visual inspection of the simulated trajectories reveals that the affected residue (CYS6) flips upon modification, concomitantly causing partial unfolding of the β-strand formed by the native residue (Fig. 4). For this reason, in the case of free-energy differences only, we do not include the results for CYS6.

Small error bars, however, must be distinguished from accuracy. In addition to potential sampling issues (i.e., apparent convergence because of local sampling), the latter depends also on the degree of systematic error of the employed method. In particular, alchemical modifications involving net-charge perturbation are a sizeable source of systematic error in free-energy calculations, because of limitations in the methodology. Even though corrections to free energies of charging could be theoretically applied to obtain more accurate and reliable results (60–62), we assume that this systematic error will approximately cancel when comparing free-energy differences from parallel legs of the thermodynamic cycle (Fig. 2). Supporting this possibility, Seeliger and de Groot have used a similar approach and shown that more than 50% of 25 free-energy differences involving changes in net charge in the case of barnase mutants exhibit values within 1 kcal/mol (< 4.18 kJ/mol) of the experiment (37). Nevertheless, this assumption may be questioned when the environment of the modified residue is very different in the two systems, such as solvent exposed in one case and buried in the other.

Effect of oxidative modifications on SOD1 homodimer and monomer stability

Oxidative damage of individual residues at the SOD1 homodimer interface results in a range of different effects (Fig. 5). In particular, the stability of the SOD1 homodimer is markedly decreased by carbonylation of LYS9 with $\Delta\Delta G^{nat\rightarrow oxi}_{mono\rightarrow dim} = 23.8 \pm 1.1$ kJ/mol (Fig. 5 *A*). This is a significant value, especially when compared with 1) the stability of the dimer itself, which was estimated to be between -50 and -60 kJ/mol (31,63); and 2) the experimentally measured destabilization effects of a number of

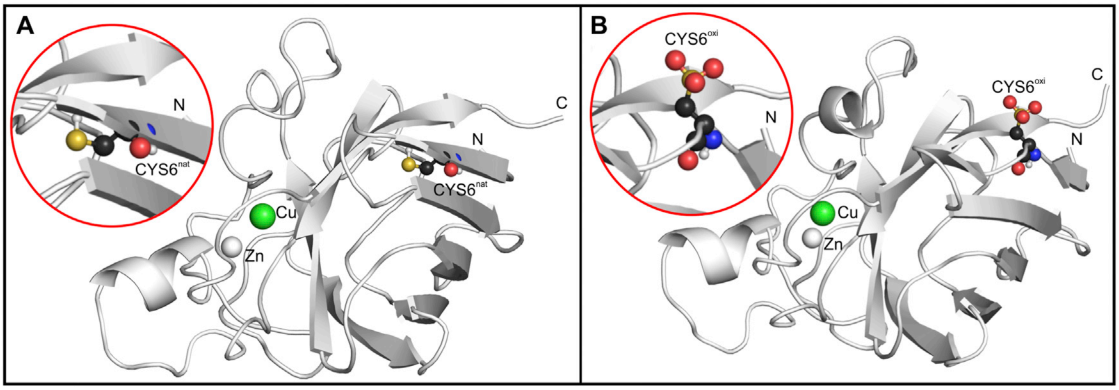

FIGURE 4 Local unfolding as a consequence of oxidative damage of CYS6. While the affected residue is buried in its native form (*A*), it flips to the protein surface and becomes solvent exposed upon oxidative modification, additionally destabilizing local β-sheet structure (*B*). *Inset*: A close-up picture of the affected residue is shown. To see this figure in color, go online.

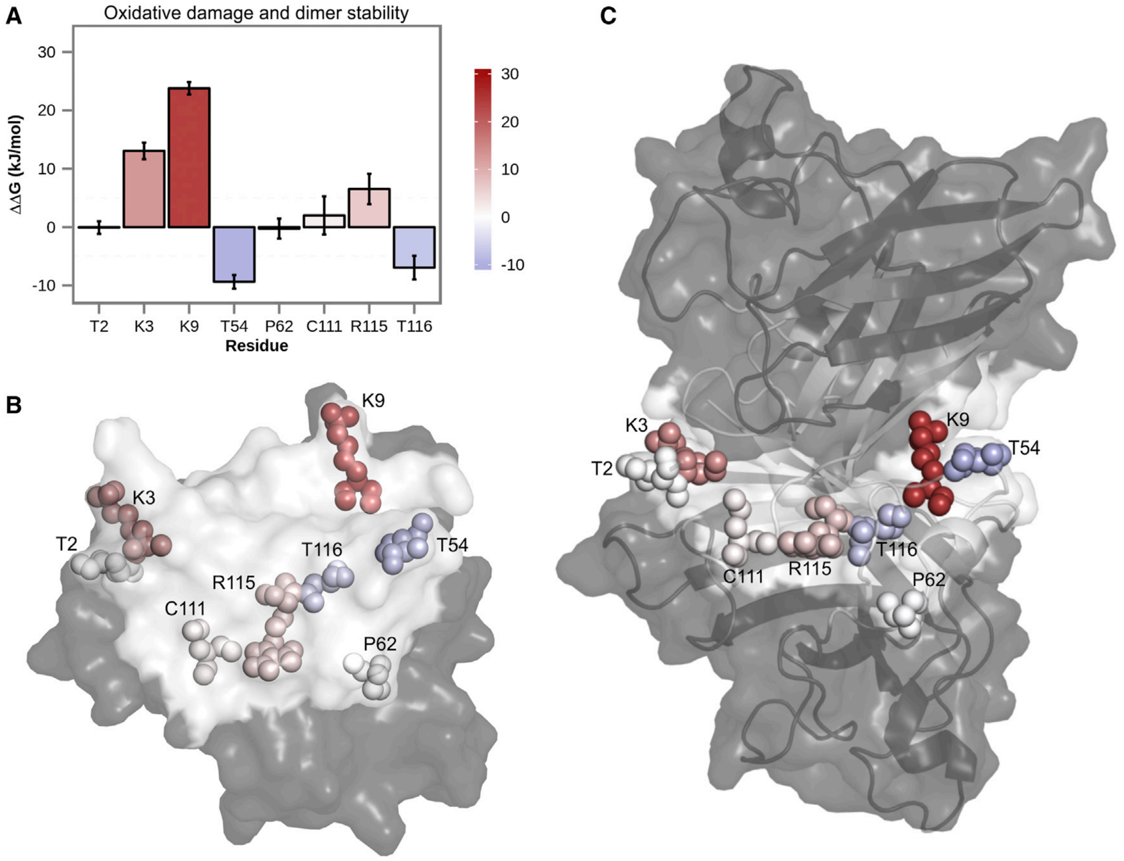

FIGURE 5 Impact on SOD1 dimer stability of oxidative damage of residues at the homodimer interface. (*A*) Changes in free energy of homodimer formation with error bars calculated by block averaging and standard propagation of error are shown. Location of the studied interface residues and effects of their oxidative modifications on the SOD1 dimer are shown in the context of (*B*) one of the monomers (view at the interface) and (*C*) SOD1 dimer. The color code for the protein structure: interface (*white*) and rest of the protein (*gray*). To see this figure in color, go online.

ALS-causing mutations with $\Delta\Delta G^{nat \rightarrow mut}_{mono \rightarrow dim}$ smaller than 5 kJ/mol (31). Furthermore, oxidatively damaged LYS3 and ARG115 residues exhibit notable destabilization as well, but to a smaller extent than LYS9 (13.1 ± 1.4 kJ/mol and 6.5 ± 2.6 kJ/mol, respectively; Fig. 5). On the other hand, threonine carbonylation stabilizes the homodimer by −9.4 ± 1.2 kJ/mol (THR54) and −6.9 ± 2.0 kJ/mol (THR116). It must be noted, however, that threonines are significantly less prone to carbonylation than other carbonylable amino acids. Lastly, oxidative modifications of THR2, PRO62, and CYS111 show little to no effect, with free-energy changes of dimer formation smaller than the calculated errors (Fig. 5). Concerning their physical location, the residues with the highest destabilizing effect upon oxidation (LYS3 and LYS9) tend to sit at the edges of the interfacial region, whereas those with the stabilizing contribution (THR54 and THR116) occupy the more central region (Fig. 5, *B* and *C*). However, considering all the examined residues, the exact location in the structure does not seem to be a main determinant of the effect of oxidation, with low-impact residues equally occupying central and peripheral regions of the interface (Fig. 5, *B* and *C*).

When it comes to the folding free energy of the SOD1 monomer, oxidative damage of ARG115, CYS111, LYS9, and LYS3 significantly affects monomer stability, decreasing it by 31.0 ± 1.3 kJ/mol, 28.7 ± 1.5 kJ/mol, 21.9 ± 0.3 kJ/mol, and 17.3 ± 0.5 kJ/mol, respectively (Fig. 6 *A*). Although such drastic destabilization effects correspond to an increase in the ratio between the unfolded and folded states of SOD1 by about four orders of magnitude, it is highly unlikely that these oxidative modifications are able to cause complete unfolding of the monomer, given that SOD1 is a hyperstable protein (64). However, they might induce partial local unfolding, as observed for CYS6 (Fig. 4), potentially leading to the formation of insoluble aggregates and consequently cytotoxicity, a mechanism already identified for SOD1 and other well-structured polypeptides involved in protein deposition disorders (65). In contrast, carbonylation modifications of the remaining residues only marginally alter monomer stability, with all of them invariably having destabilizing effects (Fig. 6 *A*). Again, the residues whose oxidation leads to the strongest destabilizing effects tend to be located at the periphery of the interfacial region (Fig. 6 *B*).

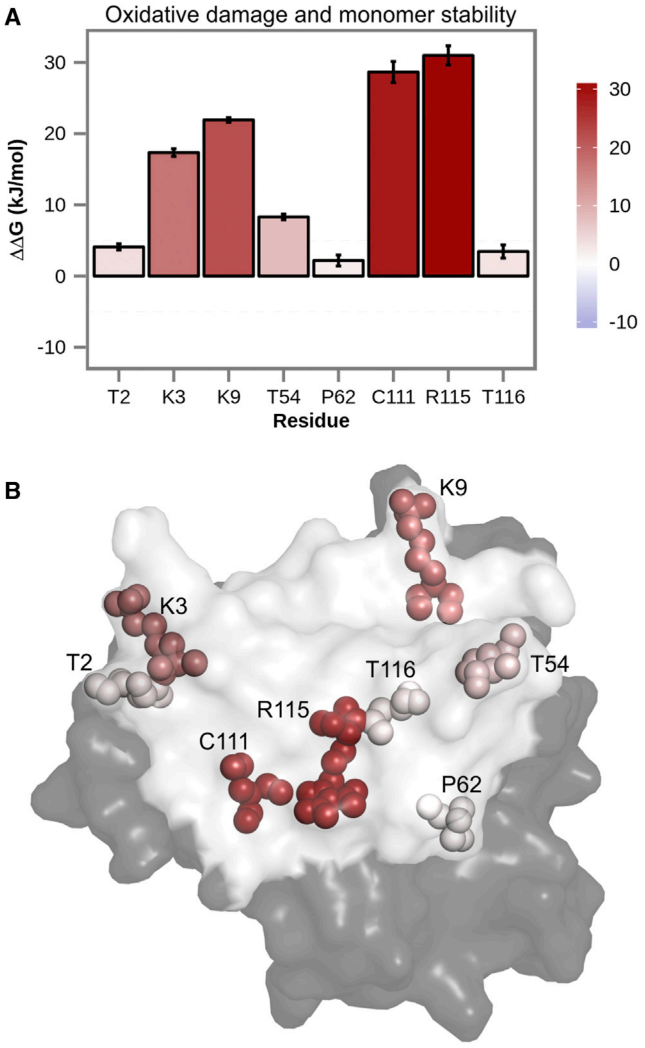

FIGURE 6 Impact on SOD1 monomer stability of oxidative damage of residues at the homodimer interface. (*A*) Changes in free energy of folding with error bars calculated by block averaging and propagation of error are shown. (*B*) Location of the studied interface residues and effects of their oxidative modifications on the SOD1 monomer (view at the interface) are shown. The color code for the protein structure: interface (*white*) and rest of the protein (*gray*). To see this figure in color, go online.

Oxidation-induced perturbation of local structure and interactions at the dimer interface

Oxidative modifications do not appear to greatly affect the local structure at the SOD1 dimer interface. In particular, the root-mean-square fluctuations (RMSF) of individual residues in native simulations are, by and large, indistinguishable from those calculated for the oxidatively damaged SOD1 (Fig. 7 *A*). Expectedly, interfacial amino acids show more flexibility in the free monomer, with average RMSF values ranging from 0.05 to 0.14 nm (Fig. 7 *A*, *left*), than in the homodimer, with average RMSF values not exceeding 0.1 nm (Fig. 7 *A*, *right*). Similarly, the root-mean-square deviations from the original experimental 3KH4 structure of the SOD1 dimer (51) show average values between 0.1 and 0.12 nm for both native and oxidatively modified variants (Fig. 7 *B*), regardless of whether one looks at deviations in the reference frame of individual monomers (*left*) or a complete dimer (*right*). This clearly indicates that the global structure of the enzyme remains intact in either case.

When it comes to the potential energy of interaction between a residue and its surrounding, oxidative damage results in a major difference for a number of residues. The highest impact is observed for CYS111, with the absolute value of the average interaction energy in the monomer and homodimer forms increasing by more than threefold. A similar effect is observed for CYS6 (Fig. 7 *C*, *left and right*). Conversely, the interaction energy of LYS3, LYS9, and ARG115 drops by more than half upon oxidation. These changes are consistent with the introduction (CYS oxidation) and removal (LYS and ARG carbonylation) of net charge, which, as expected, strongly affect the interaction energy. Additionally, the charge-conserving carbonylation of proline doubles its interaction energy (absolute value), whereas threonine carbonylation results in a slight decrease of the corresponding absolute values. Notably, comparison of the left and right panels in Fig. 7 *C* shows that all modifications alter the interaction energy of the SOD1 monomer in a similar fashion to the SOD1 homodimer. A difference between these results and the general effect of oxidative modifications on the free energy of monomer and/or dimer formation (Figs. 5 and 6) suggests that entropic effects play an important role in this process.

Regarding the hydrogen bonding networks, the main differences upon oxidation are seen in the cases of CYS6 and CYS111, with the total number of H-bonds triplicating or quadruplicating in both monomeric and dimeric forms (Fig. 7 *D*). This, of course, is fully consistent with the chemical differences between the cysteine and cysteic-acid side chains, the latter having two additional H-bond acceptor sites (Fig. 1). Finally, oxidation and a concomitant removal of a positive charge in the cases of lysine and arginine residues or the introduction of a negative charge in the cases of cysteine residues result in a rearrangement of charge-charge interactions of LYS3, CYS111, and ARG115 residues as evidenced in Fig. 7 *E*. In particular, the most pronounced effect is the loss of two salt bridges formed by LYS3 (with GLU21 and the C terminus) and the gain of two salt bridges formed by CYS111 (ARG115 and the N-terminus), whereas the ARG115 loses one salt bridge formed with GLU49 upon oxidative damage. On the other hand, CYS6 and LYS9 do not participate strongly in such local interactions in the native state. Rather, the drastic change in their interaction energy (comparable with other charge-changing modifications) coincides with the local unfolding in the case of CYS6 and major dimer destabilization in the case of LYS9.

DISCUSSION

In contrast to previous studies focusing primarily on the oxidative damage of the metal-binding histidine residues

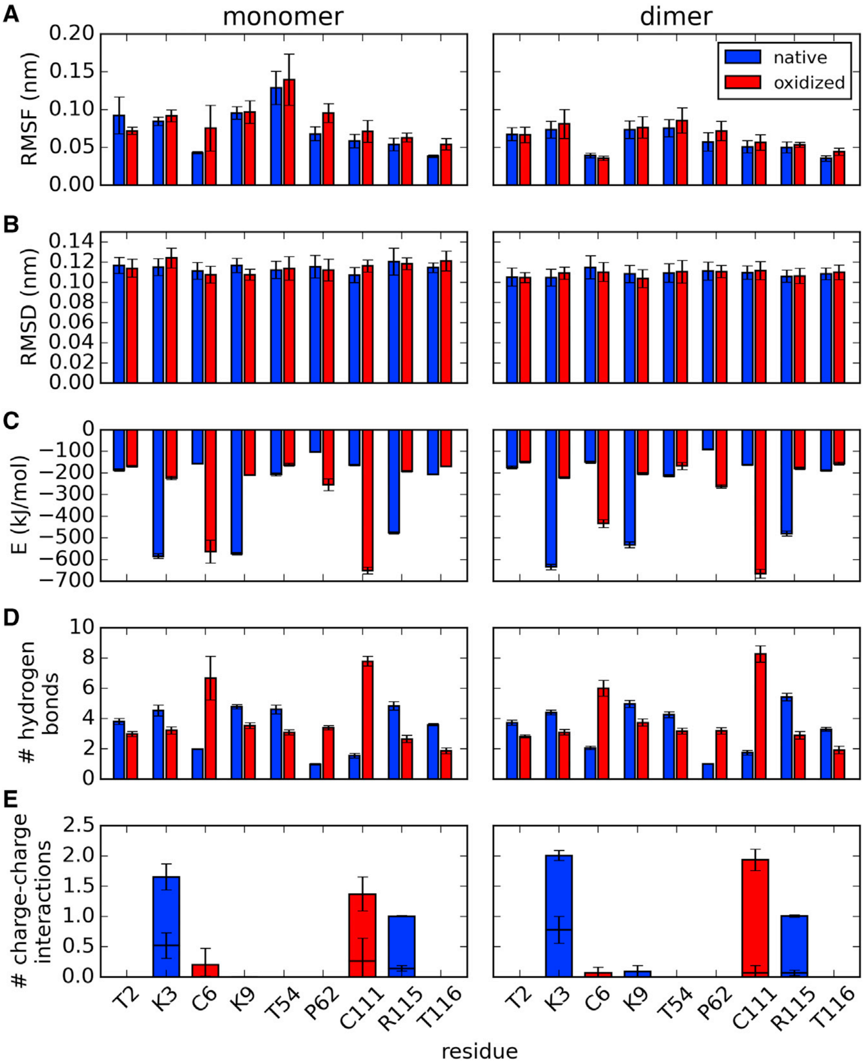

FIGURE 7 Microscopic analysis of the effects of oxidative damage on residues at the SOD1-homodimer interface: (*A*) root-mean-square fluctuations averaged over all atoms of a given amino acid; (*B*) root-mean-square deviation from the native structure; (*C*) interaction energy between a given residue and its surrounding as defined by the force field and simulation parameters used; (*D*) number of hydrogen bonds formed by a given residue; and (*E*) number of favorable (positive-negative) charge-charge interactions between a given residue and its surrounding in the native state, within 0.3 nm (*first bar*) and 0.6 nm (*second bar*). The bars in A through D represent averages over native and oxidized end-state simulations, whereas error bars in all panels show the standard deviation. To see this figure in color, go online.

in SOD1 and/or isolated cysteine residues (17–23), in this study we have systematically investigated the impact of carbonylation of all carbonylable amino acids at the SOD1 dimer interface together with that of the oxidation of interfacial cysteines. Our results show that the majority of the nine interfacial oxidative modifications in our study destabilize either the SOD1 homodimer, the monomer, or both. Specifically, oxidation of several residues significantly destabilizes both homodimeric and monomeric forms of SOD1 (carbonylation of LYS3, LYS9, and ARG115), that of other residues destabilizes only the monomeric form (carbonylation of THR54 or oxidation of CYS111), whereas in some cases oxidative damage can actually stabilize the dimer, while destabilizing the monomer (carbonylation of THR54 and THR116). Taken together, these results, support the idea that oxidative stress might lead to folding defects and impaired dimerization, which can result in an increased aggregation propensity of SOD1, cytotoxicity, and associated disorders such as ALS. Moreover, these results support the proposal (17–23) that an increased level of oxidative damage of SOD1, triggered by age, may be a key element in ALS development, with SOD1 mutations linked to familial ALS being only an additional factor making the protein more likely to become cytotoxic and increasing the probability of an early onset of the disease. These speculations are further supported by several other lines of evidence: 1) familial ALS is a late-onset disease, suggesting that other cause(s) in addition to the reported SOD1 mutations may be required for disease development; 2) as repeatedly shown, SOD1 is involved in sporadic ALS as well (66); 3) increased oxidative stress induces SOD1 aggregation both in vitro and in vivo (17,19); 4) oxidative modifications increase protein aggregation propensity and this is particularly true for carbonylation, which can drastically promote aggregation propensity even at low concentrations (6); and 5) one of the predominant determinants of longevity appears to be the resistance of the proteome integrity and protein stability to oxidative stress, as recently shown (67,68).

Our study provides a microscopic picture of the effects of oxidative damage at the SOD1 homodimer interface together with quantitative estimates of the associated impact at the level of monomer folding/dimerization thermodynamics. Importantly, we could show that oxidation of even single residues may in some cases result in a destabilization in excess of 20 kJ/mol (e.g., that of LYS9 in the case of dimerization free energy or LYS9, CYS111, and ARG115 in the case of monomer folding free energy). Given that the dimerization free energy of SOD1 has been experimentally estimated to be between −50 to −60 kJ/mol (31,63), these values suggest that two to three oxidation events at the interface may be sufficient to lead to dimer dissociation, and even single hits may significantly destabilize it. It should be emphasized, however, that our free-energy estimates critically depend on both the quality of the force field used in our simulations and the associated degree of sampling. In particular, medium-scale or large-scale conformational changes that may accompany oxidative modifications cannot be captured by our approach, which could potentially lead to inaccuracies in calculated free-energy differences. The reported values should be seen, therefore, as qualitative predictions, which can only be fully tested and verified in an experiment. Finally, five of the nine studied modifications involve net charge perturbation, potentially also affecting the quantitative aspects of the estimated changes in SOD1 monomer and dimer stability. These caveats notwithstanding, we argue that the calculated data support the qualitative interpretations given, considering the following: 1) similar methods to the one used in this study have been successfully applied to predict changes in protein stability (37–41); 2) we use long simulation time for each alchemical modification (> 100 ns per modification), arguably achieving good convergence; 3) we employ a state-of-the-art force field (55,56), which was parameterized to accurately capture the hydration free energy of amino-acid side chains, arguably one of the most important determinants of protein folding and protein-protein interactions; 4) we validate our approach against experimental data for three ALS-related mutations affecting residues on the homodimer interface (A4V, C6A, and I113T) with the average deviation of 4.7 kJ/mol from experiment (see Supporting Material for more details); 5) the majority of the estimated changes in free energy consistently point in the direction of destabilization, with a sizeable fraction showing drastic effects and the most extreme examples in excess of 10RT at room temperature; and 6) the obtained $\Delta\Delta G$ values fall within a similar range as those obtained also computationally for different SOD1 mutants.

In particular, Khare and Dokholyan have carried out a computational analysis of 75 different SOD1 mutants implicated in familial ALS and their impact on dimer association thermodynamics (69) and found that 70 of these lead to either decreased dimer stability or increased dimer dissociation or both, whereas 4 of them lead to decreased monomer stability. Importantly, the bulk of mutants resulted in free-energy values of destabilization of 20 kJ/mol or less. Similarly, Das and Plotkin have used steered MD simulations to analyze the impact of 21 different mutations or cysteine oxidation events on SOD1 metal affinity, dimer stability, and mechanical malleability (70). In their study, the destabilizing effect of most mutations was estimated to be between 20 and 40 kJ/mol. When compared with our results, these values suggest that oxidative "mutations" are on average as potent in destabilizing the SOD1 monomer/dimer as the known mutations implicated in familial ALS. Considering that ~90% of all ALS cases belong to the sporadic category, it is an important message of our study that oxidative damage of even individual residues may be comparable, in terms of thermodynamic impact, to the more widely studied familial ALS mutants.

CONCLUSIONS

Using MD simulations in combination with extensive free-energy calculations, to the best of our knowledge, we provide the first-ever quantitative predictions of changes in stability of SOD1 upon several relevant oxidative modifications. Our results show that oxidative damage of even single SOD1 residues can drastically destabilize both its homodimer and monomer structures, supporting a long-standing hypothesis that age-related increase in oxidative stress may trigger ALS, with the mutations in SOD1 gene being an additional factor in disease development. Our analysis provides a quantitative, microscopically detailed framework for interpreting extant experimental data and guiding future studies on molecular mechanisms behind ALS. In summary, this study directly links microscopic-level site-specific effects of age-related oxidative modifications and SOD1 monomer and homodimer destabilization, a presumable cause of ALS. We hope that our results will inspire and help the design of experimental studies addressing the molecular mechanisms connecting SOD1 oxidation and the formation of aggregates in the context of this disease.

SUPPORTING MATERIAL

Supporting discussion and one figure are available at http://www.biophysj.org/biophysj/supplemental/S0006-349516)30046-7.

AUTHOR CONTRIBUTIONS

D.P., X.D., and B.Z. conceived and designed the experiments. D.P. performed the experiments and analyzed the data. D.P., X.D., and B.Z. wrote the manuscript.

ACKNOWLEDGMENTS

We thank members of the Laboratory of Computational Biophysics at MFPL for useful advice and assistance.

This work was supported in part by the Austrian Science Fund FWF (START grant Y 514-B11 to B.Z., http://www.fwf.ac.at/), European Research Council (ERC Starting Independent grant 279408 to B.Z.,

http://erc.europa.eu/), and the HPC-EUROPA2 project with the support of the European Commission—Capacities Area—Research Infrastructures (project no. 1036 to D.P., http://www.hpc-europa.eu/).

SUPPORTING CITATIONS

References (71,72) appear in the Supporting Material.

REFERENCES

1. Levine, R. L., and E. R. Stadtman. 2001. Oxidative modification of proteins during aging. *Exp. Gerontol.* 36:1495–1502.
2. Nyström, T. 2005. Role of oxidative carbonylation in protein quality control and senescence. *EMBO J.* 24:1311–1317.
3. Dalle-Donne, I., R. Rossi, ..., A. Milzani. 2006. Biomarkers of oxidative damage in human disease. *Clin. Chem.* 52:601–623.
4. Davies, K. J. 2001. Degradation of oxidized proteins by the 20S proteasome. *Biochimie.* 83:301–310.
5. Grune, T., T. Jung, ..., K. J. Davies. 2004. Decreased proteolysis caused by protein aggregates, inclusion bodies, plaques, lipofuscin, ceroid, and 'aggresomes' during oxidative stress, aging, and disease. *Int. J. Biochem. Cell Biol.* 36:2519–2530.
6. Petrov, D., and B. Zagrovic. 2011. Microscopic analysis of protein oxidative damage: effect of carbonylation on structure, dynamics, and aggregability of villin headpiece. *J. Am. Chem. Soc.* 133:7016–7024.
7. Andersen, J. K. 2004. Oxidative stress in neurodegeneration: cause or consequence? *Nat. Med.* 10 (*Suppl.*):S18–S25.
8. Hand, C. K., and G. A. Rouleau. 2002. Familial amyotrophic lateral sclerosis. *Muscle Nerve.* 25:135–159.
9. Barber, S. C., and P. J. Shaw. 2010. Oxidative stress in ALS: key role in motor neuron injury and therapeutic target. *Free Radic. Biol. Med.* 48:629–641.
10. Ince, P. G., J. R. Highley, ..., P. J. Shaw. 2011. Molecular pathology and genetic advances in amyotrophic lateral sclerosis: an emerging molecular pathway and the significance of glial pathology. *Acta Neuropathol.* 122:657–671.
11. Abel, O., J. F. Powell, ..., A. Al-Chalabi. 2012. ALSoD: a user-friendly online bioinformatics tool for amyotrophic lateral sclerosis genetics. *Hum. Mutat.* 33:1345–1351.
12. Kato, S., M. Takikawa, ..., E. Ohama. 2000. New consensus research on neuropathological aspects of familial amyotrophic lateral sclerosis with superoxide dismutase 1 (SOD1) gene mutations: inclusions containing SOD1 in neurons and astrocytes. *Amyotroph. Lateral Scler. Other Motor Neuron Disord.* 1:163–184.
13. Liu, H.-N., T. Sanelli, ..., J. Robertson. 2009. Lack of evidence of monomer/misfolded superoxide dismutase-1 in sporadic amyotrophic lateral sclerosis. *Ann. Neurol.* 66:75–80.
14. Forsberg, K., P. M. Andersen, ..., T. Brännström. 2011. Glial nuclear aggregates of superoxide dismutase-1 are regularly present in patients with amyotrophic lateral sclerosis. *Acta Neuropathol.* 121:623–634.
15. Bruijn, L. I., M. K. Houseweart, ..., D. W. Cleveland. 1998. Aggregation and motor neuron toxicity of an ALS-linked SOD1 mutant independent from wild-type SOD1. *Science.* 281:1851–1854.
16. Johnston, J. A., M. J. Dalton, ..., R. R. Kopito. 2000. Formation of high molecular weight complexes of mutant Cu, Zn-superoxide dismutase in a mouse model for familial amyotrophic lateral sclerosis. *Proc. Natl. Acad. Sci. USA.* 97:12571–12576.
17. Oeda, T., S. Shimohama, ..., N. Ishii. 2001. Oxidative stress causes abnormal accumulation of familial amyotrophic lateral sclerosis-related mutant SOD1 in transgenic *Caenorhabditis elegans*. *Hum. Mol. Genet.* 10:2013–2023.
18. Rakhit, R., P. Cunningham, ..., A. Chakrabartty. 2002. Oxidation-induced misfolding and aggregation of superoxide dismutase and its implications for amyotrophic lateral sclerosis. *J. Biol. Chem.* 277:47551–47556.
19. Rakhit, R., J. P. Crow, ..., A. Chakrabartty. 2004. Monomeric Cu,Zn-superoxide dismutase is a common misfolding intermediate in the oxidation models of sporadic and familial amyotrophic lateral sclerosis. *J. Biol. Chem.* 279:15499–15504.
20. Valentine, J. S., P. A. Doucette, and S. Zittin Potter. 2005. Copper-zinc superoxide dismutase and amyotrophic lateral sclerosis. *Annu. Rev. Biochem.* 74:563–593.
21. Kabashi, E., P. N. Valdmanis, ..., G. A. Rouleau. 2007. Oxidized/misfolded superoxide dismutase-1: the cause of all amyotrophic lateral sclerosis? *Ann. Neurol.* 62:553–559.
22. Rotunno, M. S., and D. A. Bosco. 2013. An emerging role for misfolded wild-type SOD1 in sporadic ALS pathogenesis. *Front. Cell. Neurosci.* 7:253.
23. Martins, D., and A. M. English. 2014. SOD1 oxidation and formation of soluble aggregates in yeast: relevance to sporadic ALS development. *Redox Biol.* 2:632–639.
24. Kurahashi, T., A. Miyazaki, ..., M. Isobe. 2001. Extensive investigations on oxidized amino acid residues in H_2O_2-treated Cu,Zn-SOd protein with LC-ESI-Q-TOF-MS, MS/MS for the determination of the copper-binding site. *J. Am. Chem. Soc.* 123:9268–9278.
25. Banci, L., I. Bertini, ..., J. P. Whitelegge. 2007. Metal-free superoxide dismutase forms soluble oligomers under physiological conditions: a possible general mechanism for familial ALS. *Proc. Natl. Acad. Sci. USA.* 104:11263–11267.
26. Mulligan, V. K., A. Kerman, ..., A. Chakrabartty. 2012. Early steps in oxidation-induced SOD1 misfolding: implications for non-amyloid protein aggregation in familial ALS. *J. Mol. Biol.* 421:631–652.
27. Guareschi, S., E. Cova, ..., P. Pasinelli. 2012. An over-oxidized form of superoxide dismutase found in sporadic amyotrophic lateral sclerosis with bulbar onset shares a toxic mechanism with mutant SOD1. *Proc. Natl. Acad. Sci. USA.* 109:5074–5079.
28. Choi, J., H. D. Rees, ..., L. Li. 2005. Oxidative modifications and aggregation of Cu,Zn-superoxide dismutase associated with Alzheimer and Parkinson diseases. *J. Biol. Chem.* 280:11648–11655.
29. van Gunsteren, W. F., D. Bakowies, ..., H. B. Yu. 2006. Biomolecular modeling: goals, problems, perspectives. *Angew. Chem. Int. Ed. Engl.* 45:4064–4092.
30. Best, R. B. 2012. Atomistic molecular simulations of protein folding. *Curr. Opin. Struct. Biol.* 22:52–61.
31. Vassall, K. A., P. B. Stathopulos, ..., E. M. Meiering. 2006. Equilibrium thermodynamic analysis of amyotrophic lateral sclerosis-associated mutant apo Cu,Zn superoxide dismutases. *Biochemistry.* 45:7366–7379.
32. Rumfeldt, J. A. O., P. B. Stathopulos, ..., E. M. Meiering. 2006. Mechanism and thermodynamics of guanidinium chloride-induced denaturation of ALS-associated mutant Cu,Zn superoxide dismutases. *J. Mol. Biol.* 355:106–123.
33. Rohl, C. A., C. E. M. Strauss, ..., D. Baker. 2004. Protein structure prediction using Rosetta. *Methods Enzymol.* 383:66–93.
34. Capriotti, E., P. Fariselli, and R. Casadio. 2005. I-Mutant2.0: predicting stability changes upon mutation from the protein sequence or structure. *Nucleic Acids Res.* 33:W306–W310.
35. Benedix, A., C. M. Becker, ..., R. A. Böckmann. 2009. Predicting free energy changes using structural ensembles. *Nat. Methods.* 6:3–4.
36. Potapov, V., M. Cohen, and G. Schreiber. 2009. Assessing computational methods for predicting protein stability upon mutation: good on average but not in the details. *Protein Eng. Des. Sel.* 22:553–560.
37. Seeliger, D., and B. L. de Groot. 2010. Protein thermostability calculations using alchemical free energy simulations. *Biophys. J.* 98:2309–2316.
38. Lin, Z., J. Kornfeld, ..., W. F. van Gunsteren. 2010. Prediction of folding equilibria of differently substituted peptides using one-step perturbation. *J. Am. Chem. Soc.* 132:7276–7278.

39. Lin, Z., and W. F. van Gunsteren. 2013. Combination of enveloping distribution sampling (EDS) of a soft-core reference-state Hamiltonian with one-step perturbation to predict the effect of side chain substitution on the relative stability of right- and left-helical folds of β-peptides. *J. Chem. Theory Comput.* 9:126–134.

40. Song, X., Y. Wang, ..., L. Yao. 2013. Engineering a more thermostable blue light photo receptor *Bacillus subtilis* YtvA LOV domain by a computer aided rational design method. *PLoS Comp. Biol.* 9:e1003129.

41. Lin, Z. X., C. Necula, and W. F. van Gunsteren. 2014. Using enveloping distribution sampling to compute the folding free enthalpy of a beta-peptide with a very unstable folded conformation in solution: the advantage of focused sampling using EDS. *Chem. Phys.* 428:156–163.

42. Beveridge, D. L., and F. M. DiCapua. 1989. Free energy via molecular simulation: applications to chemical and biomolecular systems. *Annu. Rev. Biophys. Biophys. Chem.* 18:431–492.

43. Straatsma, T. P., and J. A. McCammon. 1992. Computational alchemy. *Annu. Rev. Phys. Chem.* 43:407–435.

44. Peter, C., C. Oostenbrink, ..., W. F. van Gunsteren. 2004. Estimating entropies from molecular dynamics simulations. *J. Chem. Phys.* 120:2652–2661.

45. de Ruiter, A., and C. Oostenbrink. 2011. Free energy calculations of protein-ligand interactions. *Curr. Opin. Chem. Biol.* 15:547–552.

46. Shirts, M. R., and V. S. Pande. 2005. Comparison of efficiency and bias of free energies computed by exponential averaging, the Bennett acceptance ratio, and thermodynamic integration. *J. Chem. Phys.* 122:144107.

47. Bruckner, S., and S. Boresch. 2011. Efficiency of alchemical free energy simulations. I. A practical comparison of the exponential formula, thermodynamic integration, and Bennett's acceptance ratio method. *J. Comput. Chem.* 32:1303–1319.

48. Bruckner, S., and S. Boresch. 2011. Efficiency of alchemical free energy simulations. II. Improvements for thermodynamic integration. *J. Comput. Chem.* 32:1320–1333.

49. Berlett, B. S., and E. R. Stadtman. 1997. Protein oxidation in aging, disease, and oxidative stress. *J. Biol. Chem.* 272:20313–20316.

50. Petrov, D., C. Margreitter, ..., B. Zagrovic. 2013. A systematic framework for molecular dynamics simulations of protein post-translational modifications. *PLoS Comput. Biol.* 9:e1003154.

51. Gazdag, E. M., I. C. Cirstea, ..., K. Alexandrov. 2010. Purification and crystallization of human Cu/Zn superoxide dismutase recombinantly produced in the protozoan *Leishmania tarentolae*. *Acta Crystallogr. Sect. F Struct. Biol. Cryst. Commun.* 66:871–877.

52. Berendsen, H. J. C., J. P. M. Postma, ..., J. Hermans. 1981. Interaction Models for Water in Relation to Protein Hydration. Reidel, Dordrecht, Netherlands.

53. Boned, R., W. F. van Gunsteren, and X. Daura. 2008. Estimating the temperature dependence of peptide folding entropies and free enthalpies from total energies in molecular dynamics simulations. *Chemistry.* 14:5039–5046.

54. Hess, B., C. Kutzner, ..., E. Lindahl. 2008. GROMACS 4: algorithms for highly efficient, load-balanced, and scalable molecular simulation. *J. Chem. Theory Comput.* 4:435–447.

55. Oostenbrink, C., A. Villa, ..., W. F. van Gunsteren. 2004. A biomolecular force field based on the free enthalpy of hydration and solvation: the GROMOS force-field parameter sets 53A5 and 53A6. *J. Comput. Chem.* 25:1656–1676.

56. Schmid, N., A. P. Eichenberger, ..., W. F. van Gunsteren. 2011. Definition and testing of the GROMOS force-field versions 54A7 and 54B7. *Eur. Biophys. J.* 40:843–856.

57. Berendsen, H. J. C., J. P. M. Postma, ..., J. R. Haak. 1984. Molecular dynamics with coupling to an external bath. *J. Chem. Phys.* 81:3684–3690.

58. Beutler, T. C., A. E. Mark, ..., W. F. van Gunsteren. 1994. Avoiding singularities and numerical instabilities in free energy calculations based on molecular simulations. *Chem. Phys. Lett.* 222:529–539.

59. Margreitter, C., D. Petrov, and B. Zagrovic. 2013. Vienna-PTM web server: a toolkit for MD simulations of protein post-translational modifications. *Nucleic Acids Res.* 41:W422–W426.

60. Hunenberger, P., and M. Reif. 2011. Single-ion solvation: experimental and theoretical approaches to elusive thermodynamic quantities. *In* Single-Ion Solvation: Experimental and Theoretical Approaches to Elusive Thermodynamic Quantities. J. Hirst, editor. Royal Society of Chemistry, London, pp. 1–664.

61. Rocklin, G. J., D. L. Mobley, ..., P. H. Hünenberger. 2013. Calculating the binding free energies of charged species based on explicit-solvent simulations employing lattice-sum methods: an accurate correction scheme for electrostatic finite-size effects. *J. Chem. Phys.* 139:184103.

62. Reif, M. M., and C. Oostenbrink. 2014. Net charge changes in the calculation of relative ligand-binding free energies via classical atomistic molecular dynamics simulation. *J. Comput. Chem.* 35:227–243.

63. Lindberg, M. J., J. Normark, ..., M. Oliveberg. 2004. Folding of human superoxide dismutase: disulfide reduction prevents dimerization and produces marginally stable monomers. *Proc. Natl. Acad. Sci. USA.* 101:15893–15898.

64. Forman, H. J., and I. Fridovich. 1973. On the stability of bovine superoxide dismutase. The effects of metals. *J. Biol. Chem.* 248:2645–2649.

65. Chiti, F., and C. M. Dobson. 2009. Amyloid formation by globular proteins under native conditions. *Nat. Chem. Biol.* 5:15–22.

66. Gagliardi, S., E. Cova, ..., C. Cereda. 2010. SOD1 mRNA expression in sporadic amyotrophic lateral sclerosis. *Neurobiol. Dis.* 39:198–203.

67. Pérez, V. I., R. Buffenstein, ..., A. Chaudhuri. 2009. Protein stability and resistance to oxidative stress are determinants of longevity in the longest-living rodent, the naked mole-rat. *Proc. Natl. Acad. Sci. USA.* 106:3059–3064.

68. Krisko, A., and M. Radman. 2010. Protein damage and death by radiation in *Escherichia coli* and *Deinococcus radiodurans*. *Proc. Natl. Acad. Sci. USA.* 107:14373–14377.

69. Khare, S. D., M. Caplow, and N. V. Dokholyan. 2006. FALS mutations in Cu, Zn superoxide dismutase destabilize the dimer and increase dimer dissociation propensity: a large-scale thermodynamic analysis. *Amyloid.* 13:226–235.

70. Das, A., and S. S. Plotkin. 2013. Mechanical probes of SOD1 predict systematic trends in metal and dimer affinity of ALS-associated mutants. *J. Mol. Biol.* 425:850–874.

71. Svensson, A. K. E., O. Bilsel, ..., C. R. Matthews. 2010. Metal-free ALS variants of dimeric human Cu,Zn-superoxide dismutase have enhanced populations of monomeric species. *PLoS One.* 5:e10064.

72. Lindberg, M. J., L. Tibell, and M. Oliveberg. 2002. Common denominator of Cu/Zn superoxide dismutase mutants associated with amyotrophic lateral sclerosis: decreased stability of the apo state. *Proc. Natl. Acad. Sci. USA.* 99:16607–16612.

Biophysical Journal, Volume *110*

Supplemental Information

Effect of Oxidative Damage on the Stability and Dimerization of Superoxide Dismutase 1

Drazen Petrov, Xavier Daura, and Bojan Zagrovic

Biophysical Journal

Supporting Material

Effect of Oxidative Damage on the Stability and Dimerization of Superoxide Dismutase 1

D. Petrov,[1] X. Daura,[2,3] and B. Zagrovic[1,*]

[1]Department of Structural and Computational Biology, Max F. Perutz Laboratories, University of Vienna, Vienna, Austria; [2]Institute of Biotechnology and Biomedicine, Universitat Autònoma de Barcelona, Bellaterra, Spain; and [3]Catalan Institution for Research and Advanced Studies (ICREA), Barcelona, Spain

Comparison of calculated and experimental changes in free energy upon ALS-related mutations

Using the same approach as for oxidative modifications, we have calculated changes in free energy upon three ALS-related mutations affecting residues on the homodimer interface (A4V, C6A and I113T).

The calculated free energy changes of A4V and I133T mutations match well the experimental data with all of the changes having the correct sign and deviations from experiment exhibiting a median of 4.3 kJ/mol and an average of 4.7 kJ/mol (Figure 1). The only major outlier is seen in the case of the monomer-to-dimer transition for the A4V mutant (9 kJ/mol). Finally, C6A mutation shows marginal stabilization which is in agreement with experimental findings that this mutation does not affect the stability of SOD1 (Figure 1). Experimental data were taken from (1) (A4V), (2) (C6A) and (3) (I113T).

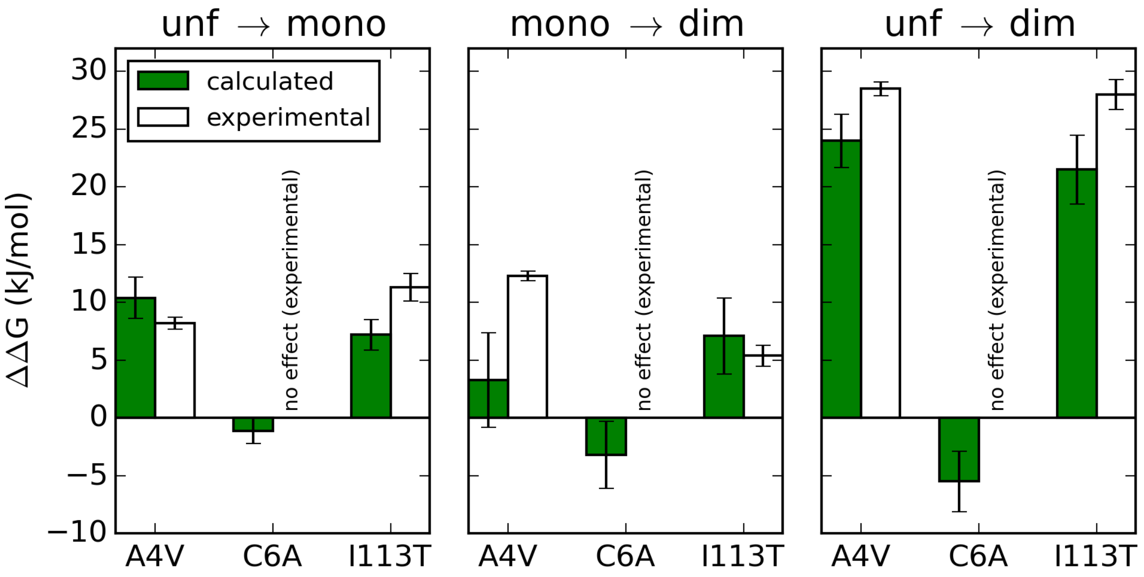

Figure 1. Comparison of the calculated and experimental changes of the free energy of SOD1 monomer folding (left), dimerization (middle) and folding and dimerization (right). Note that the free energy of folding and dimerization was calculated as: $\Delta\Delta G^{nat\to oxi}_{unf\to dim} = 2\Delta\Delta G^{nat\to oxi}_{unf\to mono} + \Delta\Delta G^{nat\to oxi}_{mono\to dim}$. "No effect" refers to experimental finding where no measureable effect of the mutation was found.

Supporting References

1. Svensson, A. K. E., O. Bilsel, C. Kayatekin, J. A. Adefusika, J. A. Zitzewitz, and C. R. Matthews. 2010. Metal-free ALS variants of dimeric human Cu,Zn-superoxide dismutase have enhanced populations of monomeric species. PLoS ONE 5:10.
2. Lindberg, M. J., L. Tibell, and M. Oliveberg. 2002. Common denominator of Cu/Zn superoxide dismutase mutants associated with amyotrophic lateral sclerosis: Decreased stability of the apo state. Proc. Natl. Acad. Sci. U.S.A. 99:16607-16612.
3. Vassall, K. A., P. B. Stathopulos, J. A. O. Rumfeldt, J. R. Lepock, and E. M. Meiering. 2006. Equilibrium thermodynamic analysis of amyotrophic lateral

sclerosis-associated mutant apo Cu,Zn superoxide dismutases. Biochemistry 45:7366-7379.
